# Are zinc-bound metallothionein isoforms (I+II and III) involved in impaired thymulin production and thymic involution during ageing?

**DOI:** 10.1186/1742-4933-1-5

**Published:** 2004-11-12

**Authors:** Eugenio Mocchegiani, Robertina Giacconi, Catia Cipriano, Elisa Muti, Nazzarena Gasparini, Marco Malavolta

**Affiliations:** 1 Immunology Ctr. (Section Nutrition, Immunity and Ageing) Res. Dept. INRCA, Ancona, Italy; 2 Immunosenescence Unit, Department of Pathobiology and Biomedical Methodologies, University of Palermo, Palermo, Italy

**Keywords:** Thymic involution, Metallothioneins, IL-6, glucocorticoids, zinc, TECs, inflammation, ageing, longevity

## Abstract

**Background:**

With advancing age, thymic efficiency shows progressive decline due to thymic involution allowing impaired cell-mediated immunity and the appearance of age-related diseases. The intrinsic cause of thymic involution is still undefined. Chronic inflammation and high glucocorticoids (GCs) may be involved. However, transgenic mice, with increased GC sensitivity and over expression of GC receptors, display delayed age-associated thymic involution. This fact suggests that other substances may affect thymic involution. Among them, both isoforms of metallothioneins (MTs) I+II and III are the major candidates because their increments leads to organ atrophy in constant stress and are induced by IL-6, which increases in ageing. Enhanced MTs in ageing allows constant sequester of zinc ions and no subsequent zinc release leading to low zinc ion bioavailability for thymic efficiency. This sequester is very limited in very old age. Thus, we have investigated the MTmRNA (I+II and III) in the thymus from young, old and very old mice.

**Methods:**

MTmRNA and IL-6mRNA (RT-PCR) in the thymus from different donors were tested. Concomitantly, TECs proliferation, zinc ion bioavailability (ratio total thymulin/active thymulin), thymulin activity and corticosterone were tested from different donors.

**Results:**

Both isoforms of MTmRNA and IL-6mRNA increase in old thymus coupled with low zinc ion bioavailability, reduced TECs proliferation, impaired thymulin activity and enhanced plasma corticosterone in comparison with young. Conversely, although the thymus is involuted in very old mice because of no changes in thymus weight in comparison to old mice, reduced MTmRNA, especially MT-I+II isoforms, and low IL6mRNA occur. Concomitantly, good zinc ion bioavailability, maintained TECs proliferation, satisfactory thymulin activity and reduced corticosterone are observed in very old mice.

**Conclusions:**

The concomitant increments by high IL-6 of both MT isoforms in the thymus from old mice may be involved in thymic involution because provoking low zinc ion bioavailability, which is relevant for thymic efficiency. By contrast, the limited increments of MTs by low IL-6 induce good zinc ion bioavailability and satisfactory thymic efficiency in very old mice. Therefore, abnormal increased MTs may provoke complete thymic involution during ageing and the possible appearance of age-related diseases. If their increments are instead limited by low inflammation, healthy ageing and longevity may be reached.

## Introduction

The thymus gland is a central lymphoid organ in which bone marrow-derived T cell precursors undergo a complex process of maturation and differentiation leading to migration of positively selected thymocytes to the T cell-dependent peripheral areas [1]. Although thymocytes proliferation and differentiation persist throughout life, they diminish with ageing. Older thymuses are significantly atrophied and have fewer thymocytes than younger ones. Therefore, the thymus undergoes an age-dependent degenerative process, which allows a progressive loss of thymocytes as well as thymic lymphoid tissue becoming involuted, atrophic and full of fat [2]. Thymic involution is particularly important in relation to immunosenescence because leading to an impaired T cell-mediated immunity with the subsequent appearance of some age-related diseases [3]. The loss of thymocytes in ageing is also due, other than to diminished size of thymic cortex, to decreased production of thymic hormonal factors, which are important for thymocytes maturation, differentiation and proliferation [4]. Thymic hormonal factors, such as thymulin, thymopentin and thymosines, are produced by the Thymic Epithelial Cells (TECs), which number and proliferation decrease in ageing together with thymocytes [5]. The following one another of thymic negative events during ageing have been attributed to concomitant increments of glucocorticoids (GCs). Specific GCs receptors are present both on thymocytes and TECs leading the thymic cells to undergo apoptosis via Fas [6]. However, it has been recently reported in transgenic mice with increased GC sensitivity and over expression of GC receptors, a delayed age-associated thymic involution when compared with wild-type mice. These mice display a higher number of thymocytes and, surprisingly, thymic apoptosis is unaffected [7]. These data suggest that endogenous GCs may not be directly involved in thymic atrophy in ageing or, at least, they may act concomitantly or synergistically with other substances. In this context, some proteins, such as zinc-bound metallothioneins (MT) (isoforms I+II and III), may be involved in age-related thymic involution for the following reasons. Firstly, MT induction is controlled by GCs and pro-inflammatory cytokines (IL-6) [8], which increase in ageing and inflammation [9]; and also high IL-6 is involved in thymic dysregulation [10]. Second, MT increases in ageing and strictly related to high IL-6 and GCs [11]. Third, high MT are harmful in immunosenescence because they sequester zinc and are unable, within old lymphocytes, in the zinc release [11], which is in turn pivotal for immune efficiency and in conferring biological activity to thymulin [12], and thymulin activity, immune efficiency and free zinc ion bioavailability decrease in ageing [13]. Fourth, concomitant increments of MT-I+II and III during persistent stress like-conditions, as it occurs in ageing [14], lead to pancreas atrophy in stressed mice [15]. Although, MT-III isoform may be only present in the brain [16], its existence also in peripheral organs has been reported [17]. Following these considerations, we have investigated the presence of MT I+II and III gene expression and zinc content in the thymus from young, old and very old mice. Concomitantly, the IL-6 gene expression and TECs number and proliferation in the thymus from different donors have been evaluated as well as thymulin activity, corticosterone and zinc plasma levels. We have chosen very old mice because the MT (I+II) gene expression is low, like in younger, allowing satisfactory peripheral immune response [11].

## Materials and Methods

### Mice

Balb/c male inbred mice were used at the age of 2–3 months (young = n.10 mice), at the age of 20 months (old = n.10 mice) and at the age of 28–30 months (very old = n.10 mice). Although the maximum thymus sizes as well as peaks in thymocytes maturation and differentiation occur at 2–4 weeks of age in mice [18], no differences in thymulin activity and TECs number exist among 1 and 2–3 months of age [19]. Therefore, the choice of young mice at 2–3 months of age for the present study is appropriate. Mice were housed in plastic non-galvanized cages (5–6 mice for cage) and fed with standard pellet food (Nossan, Italy) and tap water ad libitum. Under our housing condition, the life span of Balb/c mice was of 30 months [13]. Since about 50% of survival occurred at 20 months of age, mice at this age were considered old [13]. Mice were maintained on a 12-h light/12-h dark cycle from 7:00 a.m. to 7:00 p.m. at constant temperature (20 ± 1°C) and humidity (50 ± 5%). Mice were sacrificed under ether anaesthesia. Heparinized blood samples were collected by cardiac puncture for plasma determinations of corticosterone, thymulin and zinc. Freshly thymuses were frozen in liquid nitrogen for MT-I+II, MT-III and IL-6 mRNA expressions and for testing zinc content.

### RNA isolation and RT-PCR analysis

Total RNA was extracted from frozen thymus using Tri-Reagent according manufacture s protocol (Sigma, USA). 3 μg of RNA sample were reverse transcribed adding Olio d(T) and kept at 70°C for 10 min. dent, Raise inhibitor and MMLV reverse transcriptase were subsequently added and incubated at 37°C for 1 h. Samples were heated at 95°C to inactivate enzymes and stored at 20°C. PCRs were performed using sense and antisense primers as follows: MT-I: 5'-ATGGACCCCAACTGCTCCTGCTCCACC-3', 5'-GGGTGGAACTGTATAGGAAGACGCTGG-3' (259 bp) MT-III:5'-ATGGACCCTGAGACCTGCCCCTGTCCT-3', 5'-GGCCTCTGCCTTGGCCCCCTCTTCACC-3',(183 bp); β-actin: 5'-GGACTCCTATGTGGGTGACGAGG-3', 5'-GGGAGAGCATAGCCCTCGTAGAT-3' (366 bp); IL-6: 5'-ATGAAGTTCCTCTCTGCAAGAGACT-3', 5'-CACTAGGTTTGCCGAGTAGATCTC-3' (615 bp). Conditions for amplification were as follows: for MT-I each cycle consisted of 94°C 0.30 min, 50°C 0.30 min, 72°C 0.30 min with 30 cycles; for MT-III each cycle consisted 94°C 45 sec., 55°C 30 sec., 72°C 1.5 min with 30 cycles; for β-actin each cycle consisted 94°C 1 min, 61°C 1 min, 72°C 1 min with 24 cycles; for IL-6 each cycle consisted 94°C 1 min, 65°C 2 min, 72°C 3 min with 40 cycles. The products of amplification were size-fractionated by 2% agarose gel electrophoresis and visualized by staining with ethidium bromide. Semi-quantitative analysis of the amplified products was performed with an image analyser (Gel-doc 2000 instrument, Bio-Rad, USA). The results were evaluated as a relative unit determined by normalisation of the density of each band to that of the β-actin one. This method reflects MT protein production tested with Ag^+ ^saturation method [11].

### Plasma Active Thymulin (AT) and total thymulin (TT)

Plasma active zinc-bound thymulin (AT), as extensively described elsewhere [19], was measured using a bioassay based on the ability to restore the inhibitory effect of azathioprine on rosette formation in spleen cells from young Tx mice. Results were expressed as log_-2 _of the maximal dilution of tested plasma able to induce this phenomenon [19]. In order to avoid interference due to zinc, zinc sulphate at final concentration of 200 nM was added up to plasma samples. This fact shows the total amount of thymulin produced (active thymulin+ inactive thymulin) (TT) [19]. The ratio TT/AT is an index of zinc ion bioavailability because of strict inverse correlation between ratio TT/AT and plasma zinc levels. In particular, ratio >2 = low zinc ion bioavailability; ratio <2 = mild zinc ion bioavailability; ratio = 1 normal zinc ion bioavailability [19].

### Plasma zinc and thymus zinc content

Plasma and tissue zinc content were determined in Atomic Absorption Spectrophotometer (AAS) against zinc reference standards (Sigma USA). Plasma zinc was determined after plasma dilution 1:5. Thymus tissue (1 gr) was put in muffle furnace at 550°C overnight. The ash obtained was diluted with 3 ml of 3 N HCl and transferred to a 25 ml volumetric flask and further diluted with 3 ml of 0.36 N HCl. The determination of zinc was then performed at AAS.

### Plasma Corticosterone

Plasma corticosterone level (ng/ml) was determined by RIA rat-corticosterone-^3^H kit (ICN Biomedicals, CA, USA) and referred against a standard curve. The percentage of cross-reaction with other steroid was <0.01. The sensitivity was of 0.05 ng/ml of corticosterone.

### Immunocytochemistry studies

#### a) TECs characterization

Anti pan-cytokeratin IgG1/FITC MoAb (Sigma, USA) diluted 1/25 and anti-keratin MoAb (Sigma, USA) diluted 1/20 were used. For this latter, guinea pig IgG/FITC (Sigma, USA) diluted 1/60 was used as second antibody. These MoAbs are specific to detect TECs (cortical and medullary) [20].

#### b) TEC separation and percentage

TECs were separated with method described by Kurz et al. [20]. Briefly, the thymus from young, old and very old mice after 6 h of culture was minced into small fragments and incubated with collagenase (1 mg/ml, Sigma, USA) in PBS for 1 hr at 37°C (1 ml of collagenase solution/thymus). The choice of 6 h of culture is because the maximum thymulin production and TECs number and proliferation occurred at this time of culture in experiments of thymulin kinetic (from 1 h to 12 hrs) from young thymic cultures [21,22].

The suspension was then centrifuged (2 min, 400 g) and the pellet suspended in 1 ml of Dulbecco s modified Eagle medium/Ham s F12 medium (1:1) (DMEM/F12, Gibco, Germany). The cells were subjected to two-steps trypsin (0.1 and 0.25%, respectively) and 0.001% DNase treatment in order to avoid fibroblasts [19]. After three washes in PBS, the cells were dissociated by cautious triturating through Eppendorf tips and incubated in 3 ml of DMEM/F12 medium for 2–3 h at 37°C in humidified 5% CO_2_-atmosphere in order to make to adhere the cells. The supernatant containing unattached TEC was seeded into another plastic flask containing DMEM/F12 medium supplemented with 10% horse serum and put in culture in humidified 5% CO_2_-atmosphere. The cultures were inspected for morphologically visible fibroblasts (spindle shaped cells). In cases of significant contamination, the cells were washed with PBS and underwent again to trypsinization [20]. Separated TECs were washed three times in PBS. An aliquota (10^3^) was resuspended in 1 ml of medium and underwent to TEC percentage analysis. Percentages of separated TECs were counted in 1.000 cells at fluorescence microscope [22]. Tests were performed after pre-fixation with cold methanol in the slides. Controls were performed without the primary antibodies.

#### c) TECs proliferation

After TECs separation, another aliquota (40 × 10^3^) was resuspended in 4 ml of DMEM/F12 medium for TEC proliferation analysis, which was approached using [^3^H] thymidine incorporation using 96 microtiter plates (Nunc, Denmark). 40 × 10^3 ^TECs were put in 40 wells (100 μl/well = 103 TECs/well). 10 wells were used as young; 10 wells as old; 10 wells as very old. Concomitantly, 1 μCi [^3^H]-thymidine/well (Amersham, UK) was added. The plates were incubated in humidified 5%-CO2 atmosphere for 6 hrs. Automatic harvester collected the samples and the amount of incorporated radioactivity was determined in a liquid scintillation beta-counter (Perkin-Elmer, USA).

### Statistical analysis

Two-tailed Student s t test, and ANOVA test (one-way) evaluated differences between means. Correlations were determined by linear regression analysis by the least square method. Differences were evaluated by analysis of covariance. Differences were significant when p < 0.05.

## Results

### MT-(I+II and III) and IL-6 mRNAs and zinc content in the thymus from young, old and very old mice

Table 1 shows that MT-I+II and MT-III increase in old mice in comparison with young (p < 0.001). The same increment is also observed in very old mice as compared to young ones (p < 0.01), but at lower levels than old especially for MT-I+II. The increments of both isoforms of MT in old mice are correlated with high gene expression of IL-6 when compared to young mice (p < 0.01). The increments of IL-6 from the thymus of very old mice are lower, but still significant when compared to young (p < 0.05). Conversely, the zinc content within the thymus is very high in old mice as compared to young and very old mice (p < 0.01). Since AAS tests zinc-bound and zinc unbound [12], this last finding is not so surprising because it suggests that a large amount of zinc ions are bound to MT in the thymus from old mice. As a consequence, free zinc ions are not available for thymic efficiency in old age. Significant positive correlation exists between zinc content and MT-I+IImRNA from the thymus of young, old and very old mice (r = 0.83, p < 0.01). The thymus weight from old and very old mice is strongly reduced in comparison to young mice (p < 0.001), but with no changes between old and very old mice (Table 1).

**Table 1:** MT-I+II, MT-III, IL-6 mRNAs and zinc content in the thymus from young, old and very old mice.

**Mice**	**MT-I+II (MT-I/βactin)**	**IL-6 (IL-6/βactin)**	**MT-III (MT-III/βactin)**	**Zinc content (μg/gr.)**	**Absolute thymus weight (mg)**
**Young**	0.18 ± 0.02	0.14 ± 0.03	0.48 ± 0.02	62.3 ± 11.2	30.6 ± 5.0
**Old**	3.52 ± 0.3*	0.23 ± 0.02^§^	1.65 ± 0.03*	107.4 ± 27.5**	13.6 ± 2.0^§^
**Very old**	1.29 ± 0.6^+^	0.18 ± 0.04+	1.63 ± 0.02*	77.4 ± 8.7	15.4 ± 2.3^§^

*p < 0.001 when compared to young mice; ^+^p < 0.01 when compared to old mice;

**p < 0.01 when compared to young and very old mice; ^§^p < 0.01 when compared to young mice;

^++^p < 0.05 when compared to old mice

### Thymic efficiency, plasma zinc and Corticosterone in young, old and very old mice

Table 2 shows that thymulin activity is strongly reduced in old mice in comparison with young (p < 0.001). Thymulin activity is instead satisfactory in very old mice when compared to old ones (p < 0.05), even if its plasma value does not reach to that observed in young mice (Table 2). These data reflect the number (in percent) and the proliferation of TECs. Both TECs number and proliferation are reduced in old mice when compared to young and very old mice (p < 0.01), even if the TECs proliferation is lower in very old mice than in young ones, but still significant in comparison with old mice (p < 0.05) (Table 2). The proliferation data in young mice agree with testing TECs proliferation in pure murine TECs cell line (IT-45RI), as previously shown [21].

**Table 2:** Zinc ion bioavailability, corticosterone, thymulin and TECs number and proliferation in young, old and very old mice

**Mice**	**Thymulin activity (log_-2_)**	**AT/TT (log_-2 _(zinc ion bioavailability)**	**Corticosterone (ng/ml)**	**Plasma zinc (μg/dl)**	**% TECs**	**TECs proliferation (cpm)**
**Young**	5.5 ± 0.5	1.1 ± 0.3	153 ± 18.3	110 ± 11	53 ± 11	750 ± 25
**Old**	1.07 ± 0.3*	3.0 ± 0.3**	265 ± 16.6**	80 ± 5.7^++^	21 ± 7**	227 ± 34**
**Very old**	2.5 ± 0.3^+^	1.0 ± 0.3	180 ± 12.4^+^	87 ± 43^++^	40 ± 12^+^	450 ± 39^+^

*p < 0.001 when compared to young mice; ^+^p < 0.05 when compared to old mice;

**p < 0.01 when compared to young and very old mice; ^++^p < 0.01 when compared to young mice.

The ratio total thymulin (TT)/active thymulin (AT) represents the zinc ion bioavailability. More high is the ratio (≥ 2) less zinc ion bioavailability is present, whereas ratio < 2 or equal to 1 means satisfactory or good zinc ion bioavailability, respectively [19]. The ratio TT/AT is higher in old mice in comparison with young and very old mice (p < 0.01) (Table 2). This fact means that a good zinc ion bioavailability exists in very old mice, as in younger ones, despite plasma zinc levels are lower in very old mice than in young ones (p < 0.01) and, at the same time, not different to those observed in old mice (Table 2). With regard to plasma Corticosterone, higher values are observed in old mice when compared to young and very old mice (p < 0.01) (Table 2).

Significant inverse correlation exists between zinc and Corticosterone (r = -0.71, p < 0.01), whereas significant positive correlation exists between thymulin activity and TECs number and proliferation (r = 0.81, p < 0.01; r = 0.79, p < 0.01, respectively) from young, old and very old mice.

## Discussion

Although MT-III isoform may be present exclusively within the brain [16], some peripheral organs (testis, prostate, epididymis, tongue, ovary, uterus, stomach, heart, pancreas and seminal vesicles) may also express MT-III isoform together with MT-I+II [17]. We herein present for the first time the concomitant gene expression of MT-I+II and MT-III also in the thymus. Both isoforms of zinc-bound MT (I+II and III)mRNA increase within the thymus of old mice, but with a minor extent of MT-I+II isoform in the thymus from very old mice. Concomitantly, the gene expression of IL-6 is higher in old mice than in young and very old ones. The zinc content within the thymus is enhanced in old mice respect to young and very old mice, whereas some thymic functions (thymulin activity and TECs number and proliferation) are impaired in old mice and preserved in very old mice. These last findings regarding to zinc content and thymic efficiency seems contradictory between old and very old mice. Really, they are not contradictory. Since AAS tests zinc-bound and zinc-unbound [13], the higher zinc content in the old thymus is largely due to high zinc-bound MTs, which recall zinc from the periphery, via zinc transporters ZnT1–4 [23], and sequester a lot amount of zinc ions [22]. The inflammation provokes zinc loss with subsequent impairment of immune response [24]. Thus, such a recall and sequester by MT are due to the great inflammation by high IL-6 and GCs because zinc ions have not to be lost. But, free zinc ions are subsequently not available for thymic efficiency due to inability of MT in zinc release in constant inflammation [14]. Conversely, limited recall of zinc ions occurs in very old mice because the inflammation is less deep. As a consequence, the zinc content in the thymus from very old mice is lower and, at the same time, more free zinc ions are available for thymic efficiency by low zinc-bound MT. Anyway, the present data show that abnormal increments of both isoforms of MT are present in the atrophic thymus from old and very old mice, but with less extent of MT-I+II in very old mice.

MT-I+II and III are expressed in the brain with a balance between the two isoforms (25). When one isoform increases, the other decreases due to a possible genetic control of MRE region on the chromosome 8, which brings the two isoforms [26]. Concomitant increments of the two isoforms within the hippocampus from old rats leads to impaired number and functions of synapses coupled with low zinc ion bioavailability [27]; and synaptic function is zinc-dependent [28]. The same phenomena occur in age-related neurodegenerative diseases [29], suggesting a possible role of increased MT-I+II and III in neurodegeneration [27]. Moreover, the concomitant presence of MT isoforms provokes the atrophy of the pancreas in stressed mice [15]. Therefore, enhanced MT I+II and III in the old thymus may lead to the thymic involution and atrophy because of an unbalance between the two MT isoforms. The mechanism may be largely due to the constant sequester of zinc ions by MT (I+II and III) with no subsequent zinc release leading to low zinc ion bioavailability for thymic endocrine activity and TECs proliferation [13]. In this context, IL-6 and glucocorticoids (GCs) may play key roles because IL-6 and GCs affect MTmRNA [8] and, in turn, abnormal high GC levels are involved in thymic atrophy through the activation of GC receptors on TECs and thymocytes [6]. A lack of free zinc ions also provokes thymic atrophy [30]. We have found enhanced IL-6mRNA within the thymus from old mice. Concomitantly, strong increments of plasma corticosterone are observed. Such increments are strictly related to high MTmRNA and low zinc ion bioavailability. These findings suggest that the chronic inflammation, via IL-6 and GCs, allows high MTs induction, low zinc ion bioavailability and subsequent thymic atrophy in old mice. The thymus is obviously atrophic in very old mice. But, the MT-I+IImRNA and IL-6mRNA are lower than old mice as well as reduced corticosterone. High corticosterone provokes zinc loss by urine and faeces [31]. Corticosterone is low in very old mice (Table 2). Thus, very old mice display more free zinc ion bioavailability with subsequent more thymic efficiency and preserved TECs number and proliferation. TECs produce thymulin, a zinc-dependent thymic hormone [12]. Zinc-bound MTs transfer zinc to thymulin in TECs [32]. The less MT-I+IImRNA in very old mice may thus allow less sequester of zinc ions. Alternatively, an easier release of zinc by MT for thymic efficiency might occur due to reduced inflammation by low IL-6mRNA and corticosterone. Anyway, the thymus from very old mice is still efficient despite it is involuted because the thymus weight between old and very old mice does not change (Table 1). This means that the thymic reconstitution in old age might not be necessary because the age-related loss of thymic efficiency appears to be only quantitative and not qualitative [33]. Thus, it might be sufficient to maintain inflammatory status and MTs homeostasis below a critical threshold in order to preserve thymic efficiency. Further experiments in thymic output from very old mice and in genes involved in thymocytes maturation and differentiation (Rag 1 and Rag 2) [2] are in progress in our lab.

On the other hand, MT-ImRNA is lower in lymphocytes from human nonagenarians coupled with satisfactory thymic and peripheral immune efficiency and good zinc ion bioavailability [11]. Moreover, very old mice display still efficient thymic functions during liver regeneration after partial hepatectomy (model of acute and constant inflammation) [34]. In addition, the presence of involuted thymus in stressed MT transgenic mice [22] further supports the involvement of high MT in thymic involution during ageing.

However, the thymic involution is not irreversible phenomena because zinc treatment in old mice restores thymic efficiency with a re-growth of thymic cortex [19]. This finding suggests that in ageing the thymus is in quiescent phase, which is less deep in very old age due probably to more zinc ion bioavailability, via MTs homeostasis, and less inflammation. Indeed, zinc also affects cell cycle and, therefore, cellular proliferation [35]. Thus, the thymus from very old mice is still active and not quiescent. The satisfactory zinc ion bioavailability coupled with the maintenance of TECs proliferation in the thymus from very old mice is in line with this interpretation. This means that in vivo condition TECs from very old mice are still capable to proliferate at the occurrence, for example in presence of external noxae in order to have a sufficient thymulin production for a prompt immune response, as occurring in human centenarians, who are high responder individuals showing a great capacity in remodelling thymulin activity [36].

In conclusion, concomitant increments of zinc-bound MT-I+II and III within the thymus may lead to thymic involution in ageing because they sequester zinc and are unable in the subsequent zinc release, which is indispensable for thymic efficiency. The cause may be related to chronic inflammation by high IL-6 and GCs. Without excluding a direct role of GCs in thymic involution [6], GCs may synergistically act with MT isoforms because GCs also affect MTmRNA [8]. Less inflammation by low IL-6 and GCs in very old mice allows reduced MTmRNA with subsequent satisfactory zinc ion bioavailability and, therefore, preserved thymic efficiency.

However, if the thymic involution may be a necessary event in order to avoid autoimmune phenomena in ageing is an intriguing point to be investigated. Indeed, high IL-6 provokes an enlargement of the thymus with the appearance of autoimmune phenomena [10]. Thus, if on one hand high MTs may be of protection inducing thymic involution in order to escape autoimmune phenomena by high IL-6, on the other hand high MT are harmful because leading to low zinc ion bioavailability for thymic efficiency. However, in this context, the involvement of zinc and MT in the efficiency of extrathymic T-cell pathway [13] has to be also considered, because this pathway is prominent in ageing and autoimmunity in order to compensate thymic failure [37]. Very old mice display satisfactory thymic efficiency (present study) and good extrathymic T-cell functions [38]. Moreover, thyroid autoantibodies are rare in healthy centenarians [39]. Therefore, the thymic involution, via MTs homeostasis, has to be limited or controlled concomitantly with the appearance of efficient extrathymic T-cell functions in order to reach healthy ageing and longevity. In other words, a correct balance between thymic involution and extrathymic T-cell functions has to exist in ageing. Otherwise, a complete thymic atrophy by abnormal high MT-I+II and III, via high IL-6 may provoke continuous immune dysfunctions (thymic and extrathymic). Altered genetic controls between the two MT isoforms on MRE region of chromosome 8 may be involved, representing an interesting field of investigation in immunosenescence. Works are in progress in our lab.
